# P-327. Mitigating Colonization with Carbapenem-resistant Organisms among Neonatal Intensive Care Unit Admissions: Evaluating the Effectiveness of Infection Control Interventions

**DOI:** 10.1093/ofid/ofae631.530

**Published:** 2025-01-29

**Authors:** Fahmida Chowdhury, Gazi Md Salahuddin Mamun, Sanzida Khan, Syeda Mah-E-Muneer, Md Aminul Islam, Dilruba Ahmed, Debashis Sen, Md Golam Dostogir Harun, Lisa P Oakley, Gemma Parra, Ashley R Styczynski

**Affiliations:** icddr,b, Dhaka, Dhaka, Bangladesh; icddr,b, Dhaka, Dhaka, Bangladesh; icddr,b, Dhaka, Dhaka, Bangladesh; icddr,b, Dhaka, Dhaka, Bangladesh; icddr,b, Dhaka, Dhaka, Bangladesh; icddr,b, Dhaka, Dhaka, Bangladesh; icddr,b, Dhaka, Dhaka, Bangladesh; icddrb, Dhaka, Dhaka, Bangladesh; Centers for Disease Control and Prevention, Atlanta, GA, United States, Atlanta, Georgia; Centers for Disease Control and Prevention, Atlanta, GA, United States, Atlanta, Georgia; Centers for Disease Control and Prevention, Atlanta, GA

## Abstract

**Background:**

Antimicrobial resistance (AMR) is the global leading cause of death from infections, particularly affecting low- and middle-income countries. Colonization with carbapenem-resistant organisms (CRO) increases the risk of infections with CRO. In an interim analysis of a prospective cohort study, we found 77% (141/182) of neonates in a neonatal intensive care unit (NICU) of a tertiary-care hospital in Bangladesh were colonized with carbapenem-resistant *Klebsiella pneumoniae* (CR-Kpn), with 63% (89/141) acquiring colonization >48 hours after hospitalization. We aimed to assess the impact of infection control (IC) interventions on CRO colonization among neonates admitted to the NICU.

**Methods:**

From July 2023 to April 2024, we enrolled 604 neonates: 358 before the intervention and 246 after. Neonates were assessed for CRO colonization using rectal swabs collected on admission, days 3 and 7, and weekly thereafter. Swabs were plated on selective agar mSuperCARBA followed by confirmatory identification and susceptibility testing using VITEK® 2. Positive blood cultures collected from patients with suspected sepsis underwent testing using VITEK® 2. Our IC interventions targeted hand hygiene (HH) and environmental cleaning (EC), including staff training on proper HH techniques and more frequent cleaning of high-touch surfaces, as well as regular audits to measure compliance with HH and EC. We conducted descriptive analyses using Pearson’s Chi-square test.

**Results:**

Compliance with HH improved from 13% to 28% following the interventions, while environmental cleaning improved from 9% to 43% based on fluorescent markers. Over the same time, CRO colonization decreased from 74% (265/358) to 63% (153/246) (p=0.002) and CR-Kpn colonization decreased from 65% (232/358) to 54% (134/246) (p=0.011). Overall bacterial blood culture positivity [54% (51/95) vs. 57% (30/53), (p=0.87)] and CRO rates [13% (12/95) vs. 21% (11/53), p=0.19] remained stable at pre and post intervention respectively.

**Conclusion:**

After implementing IC interventions, we observed a decrease in CRO colonization among NICU patients, though blood culture positivity was unchanged. Results suggest that even modest improvements in IC practices may have a role in curbing CRO transmission in high-risk settings.

**Disclosures:**

**All Authors**: No reported disclosures

